# Cardiovascular Disease in the Context of Metabolic Dysfunction-Associated Steatotic Liver Disease (MASLD): A Comprehensive Narrative Review

**DOI:** 10.3390/ijms262311275

**Published:** 2025-11-21

**Authors:** Attia Mustafa, Chris Kite, Lukasz Lagojda, Alexander Dallaway, Kamaljit Kaur Chatha, Nwe Ni Than, Eva Kassi, Ioannis Kyrou, Harpal S. Randeva

**Affiliations:** 1 Warwickshire Institute for the Study of Diabetes, Endocrinology and Metabolism (WISDEM), University Hospitals Coventry and Warwickshire NHS Trust, Coventry CV2 2DX, UK; attia.mustafa@warwick.ac.uk (A.M.); c.kite2@wlv.ac.uk (C.K.); l.lagojda@sheffield.ac.uk (L.L.); alex.dallaway@wlv.ac.uk (A.D.); 2 Warwick Medical School, University of Warwick, Coventry CV4 7AL, UK; kamaljit.chatha@uhcw.nhs.uk; 3 Internal Medicine Department, Faculty of Medicine, Omar Al Mukhtar University, Al-Bayda P.O. Box 919, Libya; 4 Institute for Cardiometabolic Medicine, University Hospitals Coventry and Warwickshire NHS Trust, Coventry CV2 2DX, UK; 5 School of Health and Wellbeing, Faculty of Education, Health and Wellbeing, University of Wolverhampton, Wolverhampton WV1 1LY, UK; 6 Division of Public Health, Sport and Wellbeing, Faculty of Health, Medicine and Society, University of Chester, Chester CH1 4BJ, UK; 7 Centre for Health and Related Research (SCHARR), School of Medicine and Population Health, University of Sheffield, Sheffield S1 4DA, UK; 8 Department of Biochemistry and Immunology, University Hospitals Coventry and Warwickshire NHS Trust, Coventry CV2 2DX, UK; 9 Institute of Precision Diagnostics and Translational Medicine, University Hospitals Coventry and Warwickshire NHS Trust, Coventry CV2 2DX, UK; 10 Gastroenterology and Hepatology Department, University Hospitals Coventry and Warwickshire NHS Trust, Coventry CV2 2DX, UK; sophia.than@uhcw.nhs.uk; 11 Department of Biological Chemistry, Medical School, National and Kapodistrian University of Athens, 11527 Athens, Greece; ekassi@med.uoa.gr; 12 Endocrine Unit, 1st Department of Propaupedic Internal Medicine, Laiko Hospital, National and Kapodistrian University of Athens, 11527 Athens, Greece; 13 Aston Medical School, College of Health and Life Sciences, Aston University, Birmingham B4 7ET, UK; 14 College of Health, Psychology and Social Care, University of Derby, Derby DE22 1GB, UK; 15 Centre for Sport, Exercise and Life Sciences, Research Institute for Health & Wellbeing, Coventry University, Coventry CV1 5FB, UK

**Keywords:** metabolic dysfunction-associated steatotic liver disease, MASLD, non-alcoholic fatty liver disease, NAFLD, metabolic dysfunction-associated fatty liver disease, MAFLD, cardiovascular disease, coronary artery disease, atrial fibrillation, heart failure

## Abstract

Metabolic dysfunction-associated steatotic liver disease (MASLD) is a chronic hepatic disease with a rising global prevalence (25–38% of the general population). As a new term, MASLD was introduced in 2023 to replace the previous nomenclature of non-alcoholic fatty liver disease (NAFLD) and metabolic dysfunction-associated fatty liver disease (MAFLD). This new term/definition introduced changes in the diagnostic criteria and underscores the direct link between cardio-metabolic risk and this prevalent liver disease. In this context, the present review examines the clinical and pathophysiological links between MASLD and cardiovascular disease (CVD), providing a robust evidence synthesis of primarily systematic review data on the association between MASLD and coronary artery disease (CAD), atrial fibrillation (AF), and heart failure (HF). This association appears to be not only synergistic, but also independent of other known CVD risk factors, highlighting MASLD as a key cardio-metabolic risk factor that merits prompt diagnosis and treatment. The development of MASLD-related cardiovascular morbidity increases with the severity of the underlying hepatic pathology, particularly with progression to steatohepatitis and fibrosis. Notably, growing evidence highlights the links between MASLD and CVD through cardiac structural, electrical, and functional alterations that can progress to CAD, AF, and new-onset HF. Recognizing these links in clinical practice underscores the importance of early detection and multi-disciplinary management of MASLD to prevent disease progression and CVD complications.

## 1. Introduction

Metabolic dysfunction-associated steatotic liver disease (MASLD), previously referred to as non-alcoholic fatty liver disease (NAFLD), is a chronic liver disease with increasing prevalence worldwide [1]. Indeed, MASLD impacts approximately 25–38% of the general population, albeit with substantial geographic variations attributed, at least partly, to differences in both lifestyle and genetic factors (e.g., Latin America exhibits the highest prevalence rates, followed by the Middle East and North Africa, while Europe exhibits the lowest) [1]. The hallmark of this prevalent hepatic disease is excess accumulation of fat in hepatocytes (steatosis; presence of ≥5% steatotic hepatocytes), which may be further complicated by local inflammation (steatohepatitis) and fibrosis, potentially leading to cirrhosis [2]. Thus, the spectrum of the underlying hepatic pathology ranges from simple steatosis to steatohepatitis and fibrosis, which may further progress to cirrhosis and even hepatocellular carcinoma [3,4,5]. Due to such complications, steatotic liver disease is expected to become the leading cause of liver transplantation by 2030 [6]. Moreover, MASLD is linked to a range of extrahepatic complications, including cardiovascular disease (CVD) which is the leading mortality cause in this patient population [7,8,9]. The close links between steatosis/steatohepatitis and the metabolic syndrome, encompassing central obesity, type 2 diabetes mellitus (T2DM), hypertension, and dysregulated lipid metabolism, largely mediate this increased CVD risk [10,11]. Accordingly, growing evidence indicates that metabolic-related steatotic liver disease increases the risk of multiple cardiac complications, including coronary artery disease (CAD), atrial fibrillation (AF), and aortic valve sclerosis, as well as left ventricular hypertrophy (LVH) which is associated with the development of heart failure with preserved ejection fraction (HFpEF) [12].

**Figure 1:**
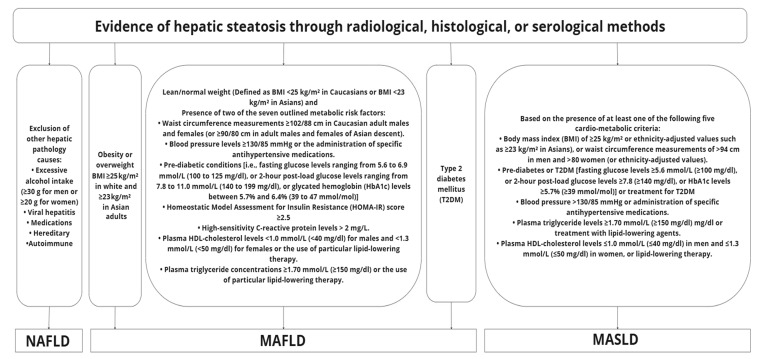
Overview of the diagnostic criteria for non-alcoholic fatty liver disease (NAFLD), metabolic dysfunction-associated fatty liver disease (MAFLD), and metabolic dysfunction-associated steatotic liver disease (MASLD) [13,15,17].

To highlight the close links between steatosis/steatohepatitis and cardio-metabolic disease, an international expert consensus introduced the term metabolic dysfunction-associated fatty liver disease (MAFLD) in 2020. MAFLD is defined based on evidence of hepatic steatosis through histological, radiological, or serological methods, combined with the presence of either overweightness/obesity status [defined in adults as body mass index (BMI) ≥ 25 kg/m^2^ in Caucasians or ≥23 kg/m^2^ in Asians] or, T2DM [13,14]. For adults with normal weight (defined as BMI < 25 kg/m^2^ in Caucasians or <23 kg/m^2^ in Asians) and steatosis, the diagnosis of MAFLD further requires at least two out of the following seven metabolic risk factors: (i) waist circumference measurements ≥ 102 and ≥88 cm in Caucasian adult males and females, respectively (or ≥90 and ≥80 cm in adult males and females of Asian descent, respectively); (ii) blood pressure ≥ 130/85 mmHg or specific antihypertensive treatment; (iii) pre-diabetic status [i.e., fasting glucose levels ranging from 5.6 to 6.9 mmol/L (100 to 125 mg/dl), or 2-h post-load glucose levels ranging from 7.8 to 11.0 mmol/L (140 to 199 mg/dl), or glycated hemoglobin (HbA1c) between 5.7% and 6.4% (39 to 47 mmol/mol)]; (iv) Homeostatic Model Assessment for Insulin Resistance (HOMA-IR) score ≥ 2.5; (v) high-sensitivity C-reactive protein (hsCRP) > 2 mg/L; (vi) plasma high-density lipoprotein (HDL) cholesterol levels < 1.0 mmol/L (< 40 mg/dl) for males and < 1.3 mmol/L (< 50 mg/dl) for females or the use of specific lipid-lowering therapy; and (vii) plasma triglyceride concentrations ≥ 1.70 mmol/L (≥ 150 mg/dl) or the use of specific lipid-lowering therapy [13,14]. In 2023, a further change to the nomenclature for NAFLD was proposed through a multi-society Delphi consensus statement, introducing the term MASLD to replace the terms NAFLD and MAFLD and discontinue the use of the term “fatty” which was considered stigmatizing for patients [15]. The diagnosis of MASLD in adults is based on the presence of at least one of the following five cardio-metabolic criteria: (i) BMI ≥ 25 kg/m^2^ (or ethnicity-adjusted BMI values, such as ≥23 kg/m^2^ in Asians), or waist circumference > 94 cm in men and > 80 cm in women (or ethnicity-adjusted waist circumference values); (ii) pre-diabetes or T2DM [fasting glucose levels ≥ 5.6 mmol/L (≥100 mg/dl), or 2-h post-load glucose levels ≥ 7.8 (≥140 mg/dl), or HbA1c ≥ 5.7% (≥39 mmol/mol)] or treatment for T2DM; (iii) blood pressure ≥ 130/85 mmHg or treatment with antihypertensive medications; (iv) plasma triglyceride levels ≥ 1.70 mmol/L (≥150 mg/dl), or treatment with lipid-lowering agents; (v) plasma HDL-cholesterol levels ≤ 1.0 mmol/L (≤40 mg/dl) in men and ≤1.3 mmol/L (≤50 mg/dl) in women, or lipid-lowering therapy [15]. An overview of the diagnostic criteria for NAFLD, MAFLD, and MASLD in adults is presented in Figure 1. For the diagnosis of MASLD, the alcohol intake should not exceed 140 g and 210 g per week for females and males, respectively, while the term metabolic- and alcohol-related/associated liver disease (MetALD) was introduced for the cases where excess alcohol intake is present along with metabolic risk factors to explain the cause of hepatic steatosis [15]. As with NAFLD, both MAFLD and MASLD represent a spectrum of hepatic pathology, from simple hepatic steatosis to metabolic-associated steatohepatitis (MASH) [16], which is the term introduced to replace non-alcoholic steatohepatitis (NASH) [17].

Contrary to NAFLD which is diagnosed based on the exclusion of other hepatic pathology causes (e.g., alcohol-associated liver disease, medication, hereditary causes, autoimmune liver conditions, or viral causes of hepatic disease) [18], the new nomenclature of MAFLD and MASLD places emphasis on the cardio-metabolic aspects of this prevalent chronic liver disease. Thus, these new terms represent a significant change in the diagnostic criteria for steatotic liver disease, which focuses the spotlight on the associated cardio-metabolic risk/diseases. Of note, an implication of the different criteria applied for the diagnosis of NAFLD, MAFLD, and MASLD, is that, despite the very high overlap between them, these terms cannot always be applied interchangeably. As such, caution is also required when interpreting existing primary research evidence which has been accumulated under one of these three terms (e.g., for patients with a NAFLD diagnosis) without a comparison to the other two [19]. To address challenges due to these different nomenclature/terms and diagnostic criteria/definitions, the present review offers a comprehensive evidence synthesis of primarily systematic review/meta-analyses data that exist on the association of all three existing terms (NAFLD, MAFLD, and MASLD) with CVD. To avoid confusion regarding the use of these terms, in the following sections, the present review applies the terms NAFLD, MAFLD, and MASLD interchangeably as NAFLD/MAFLD/MASLD regarding the general pathophysiology aspects of the disease, but, when cited data refer to a specific published study, the specific term/definition (i.e., NAFLD, MAFLD, or MASLD) used in the corresponding cited study will be applied.

## 2. Methods—Description of the Literature Search

Although not a systematic review, the present narrative review followed a predefined search strategy which was formulated and applied to identify English-published papers on MASLD or NAFLD or MAFLD and CVD. As such, our search strategy utilized relevant search terms and medical subject headings (MeSH) [20] for MASLD, NAFLD and MAFLD, as well as for CVD, atherosclerosis, acute coronary syndrome, myocardial infarction, coronary heart disease, atrial fibrillation, and heart failure. The searched databases included PubMed and Google Scholar, which were searched without a date or publication type limitation. Following removal of duplicate papers, two co-authors performed title/abstract screening, which focused predominantly, but not exclusively, on systematic review and meta-analysis papers, as well as on relevant primary research papers, that presented data on links between MASLD and/or NAFLD and/or MAFLD and CVD. The key relevant papers which were identified through this process were reviewed in full and were summarized to be included as relevant to the scope of the present narrative review, as detailed in the following sections.

## 3. NAFLD/MAFLD/MASLD and Subclinical/Clinical Atherosclerosis

Extensive research has established a direct correlation between dysregulated lipid metabolism in NAFLD/MAFLD/MASLD and atherosclerosis, which is the primary underlying cause of CVD [21]. Consistent systematic review and meta-analysis data have revealed a significant association between NAFLD and the atherogenic index of plasma (AIP; a prognostic marker for atherosclerosis in CVD) [22,23]. Furthermore, hepatic fibrosis and cirrhosis demonstrate a significant correlation with atherosclerosis progression [24,25]. Notably, Brill et al. have shown that patients with NAFLD develop atherogenic dyslipidemia even in the absence of NASH [26]. Moreover, a study in China with 2550 patients diagnosed with NAFLD showed that those with advanced hepatic fibrosis, as evaluated by the NAFLD fibrosis score (NFS), had over a 2-fold higher risk of increased carotid artery intima-media thickness (CIMT) and carotid plaque (both well-established markers of subclinical atherosclerosis) compared to those without, even after adjusting for other cardio-metabolic risk factors [27]. Further evidence has also demonstrated a correlation between NAFLD and impaired endothelial function, as well as increased arterial wall stiffness [28,29]. Additionally, a meta-analysis involving 4725 patients with NAFLD revealed a significant association between hepatic fibrosis and subclinical atherosclerosis, assessed via increased CIMT, coronary artery calcification (CAC) score, and arterial wall stiffness, with an odds ratio (OR) of 2.18 [95% confidence interval (CI): 1.62 to 2.93] [30]. Furthermore, the severity of subclinical atherosclerosis in that meta-analysis exhibited a positive correlation with the severity of hepatic fibrosis, as evidenced by comparisons between mild (OR: 1.64, 95% CI: 1.22 to 2.20) and severe hepatic fibrosis (OR: 3.42, 95% CI: 1.81 to 6.46) [30]. Further systematic review and meta-analysis data, including 42,410 participants (16,883 patients with NAFLD and 25,527 without; excluding individuals with a history of chest pain or prior CAD), showed that the NAFLD group demonstrated a higher risk of subclinical atherosclerosis, as evidenced by the CAC score, compared to those without (OR: 1.64, 95% CI: 1.42 to 1.90) [31]. This is in line with the findings of another study with 356 participants with NAFLD and 256 without, which showed a markedly higher risk of significant coronary artery stenosis in one or more arteries in the NAFLD group (84.6% vs. 64.1% of the participants with and without NAFLD, respectively; *p* < 0.001), with 68.3% of the patients with NAFLD requiring percutaneous intervention compared to 43.4% of those without (*p* < 0.001) [32]. Data from a large cross-sectional study, which included a total of 5121 individuals from the general population who underwent both abdominal ultrasound and computed tomography coronary angiography (CTCA), revealed that 38.6% of the participants had NAFLD, with those having a fatty liver index [FLI; an index which is based on BMI, waist circumference, and gamma-glutamyl transferase (GGT) and triglyceride levels]) ≥ 30 showing significantly higher proportions of atherosclerotic non-calcified plaque (OR: 1.37, 95% CI: 1.14 to 1.65; *p* = 0.001) [33]. Interestingly, regression of subclinical carotid atherosclerosis upon resolution of NAFLD over time has also been documented by another large retrospective cohort study in 8020 adult men (mean age: 49.2 years) without carotid atherosclerosis at baseline who underwent repeated health check-up examinations [34]. That study also confirmed the association of persistent NAFLD with a higher risk of subclinical carotid atherosclerosis development [34]. Furthermore, a comprehensive meta-analysis of 83 studies, including 21,458 patients with NAFLD and 32,606 controls, demonstrated a significant association between NAFLD and increased CIMT (mean difference: 0.10, 95% CI: 0.09 to 0.11; *p* < 0.00001) [35]. In addition, in the same study, a meta-analysis of 12 studies with 2646 patients with NAFLD and 2540 controls found that NAFLD was significantly associated with a 2-fold higher risk of carotid plaque formation (OR: 2.08, 95% CI: 1.52 to 2.86; *p* < 0.00001) [35]. Together, these findings underscore the role of NAFLD in promoting early atherosclerotic changes [35]. Table 1 summarizes findings from key systematic reviews and meta-analyses on NAFLD or MASLD and subclinical atherosclerosis measured by increased CIMT or presence of plaques, elevated CAC score, and heightened arterial wall stiffness [30,35,36,37,38,39,40,41]. The majority of the studies included in these analyses are classified as high quality studies, with a minority having moderate quality, supporting the robustness of the synthesized data.

Similarly to the data on the association between NAFLD and subclinical atherosclerosis, meta-analysis data by Toh et al. also showed a pooled coronary heart disease (CHD) prevalence of 44.6% (95% CI: 36.0% to 53.6%) among 67,070 patients with NAFLD, with the patients with NAFLD exhibiting significantly increased odds of CHD compared to those without (OR: 1.33, 95% CI: 1.21 to 1.45; *p* < 0.0001) [39]. Overall, in light of the evidence supporting a significant association between subclinical and clinical atherosclerosis and NAFLD/MAFLD/MASLD, these patients appear to be at higher risk of both fatal and nonfatal cardiovascular events, such as myocardial infarction (MI), angina, coronary revascularization, and stroke, which are linked to underlying coronary and carotid atherosclerotic plaques. A large nationwide cohort study in Sweden, including 10,422 biopsy-proven patients with NAFLD who were followed up for 13.6 years, showed that the patients with NAFLD had a significantly higher risk of developing CAD and stroke, with adjusted hazard ratios (HR) of 1.64 (95% CI: 1.54 to 1.75) and 1.58 (95% CI: 1.46 to 1.71), respectively [42]. Although that Swedish cohort consisted only of Caucasians, these results are also consistent with a meta-analysis by Targher et al. (34,043 individuals from a worldwide population with a 6.9-year median observation period), which revealed that patients with NAFLD have an increased risk of both fatal and nonfatal cardiovascular events, including CAD, MI, coronary revascularization interventions, and stroke (HR: 1.64, 95% CI: 1.26 to 2.13) [43]. Additionally, the severity of NAFLD was associated with an increased risk of cardiovascular events (HR: 2.58, 95% CI: 1.78 to 3.75) [43]. However, the studies included in this meta-analysis assessed NAFLD severity using various diagnostic modalities, including imaging, biomarkers, biopsy, and scoring systems, and, hence, this may introduce a bias into the results. Moreover, a meta-analysis by Yan et al. showed that, among patients with NAFLD, those with the highest NFS and fibrosis-4 score (FIB-4) values had a higher risk of cardiovascular events compared to those with lower (HR: 1.92, 95% CI: 1.50 to 2.47 vs. HR: 1.75, 95% CI: 1.53 to 2.00, respectively) [44]. Furthermore, another recent meta-analysis by Prasad et al. found that patients with NAFLD had a high risk of nonfatal cardiovascular events compared to controls (HR: 1.57, 95% CI: 1.33 to 1.85) [45]. In this meta-analysis, similarly to that by Targher et al., the risk of cardiovascular events was aggregated rather than reported individually [43,45]. When separately reporting the risk of MI and stroke in patients with NAFLD compared to those without, the meta-analysis by Alon et al. showed a significantly increased risk of both MI (OR: 1.66, 95% CI: 1.39 to 1.99) and stroke (OR: 1.41, 95% CI: 1.29 to 1.55) [46]. Of note, a meta-analysis, which investigated the MI risk in patients with a MASLD diagnosis, revealed that the MASLD group had a 1.26 times higher risk of developing MI with less than 3 years of follow-up compared to the non-MASLD group (HR: 1.26, 95% CI: 1.08 to 1.47) [47]. Alarming are also the data for relatively young patients with NAFLD, since a large meta-analysis which investigated the risk of cardiovascular events in patients with NAFLD under the age of 40 who were followed up for a period of 10.6 years (10,668,189 participants from four cohort studies; 11 datasets) revealed that these patients also had a higher CVD risk (HR: 1.63, 95% CI: 1.46 to 1.82), with higher risk for both MI (HR: 1.69, 95% CI: 1.61 to 1.78, *p* < 0.00001) and stroke (HR: 1.47, 95% CI: 1.39 to 1.55, *p* < 0.00001) [48]. Notably, a meta-analysis by Jamalinia et al. involving 18,524,532 individuals demonstrated sex-specific differences in CVD risk, with females with MAFLD showing a higher risk of fatal and non-fatal CVD events (HR: 1.59; 95% CI: 1.44 to 1.75) compared to males (HR: 1.37; 95% CI: 1.27 to 1.48) [49]. This disparity appears to be, at least partly, influenced by female-specific factors such as reproductive conditions, autoimmune disorders, breast cancer therapies, and pregnancy-related complications, including gestational diabetes, which increase the steatotic liver disease susceptibility and subsequent CVD risk [50,51,52].

Moreover, another meta-analysis, which included 29,906 individuals, demonstrated that NAFLD was associated with a significantly higher risk of angina compared to the controls, with a risk ratio (RR) of 1.45 (95% CI: 1.17 to 1.79) [53]. Within this meta-analysis, a separate analysis of 2180 patients with NAFLD and 2805 controls also revealed a significantly higher risk of developing CAD for the NAFLD group compared to the controls (HR: 1.21, 95% CI: 1.07 to 1.38) [53]. The association between NAFLD and acute coronary syndrome (ACS) has also been evaluated with meta-analysis data from 25 studies (593,635 patients with NAFLD and 4,915,788 controls), revealing a significantly higher ACS risk among individuals with NAFLD (OR: 1.95, 95% CI: 1.49 to 2.55, *p* < 0.00001) [35]. An overview of systematic review and meta-analyses data on the risk of cardiovascular events in NAFLD [35,39,43,45,49,53,54,55,56] is presented in Table 2, with a focus on fatal and/or nonfatal CVD events, including CAD, MI, coronary revascularization interventions, or stroke.

## 4. NAFLD/MAFLD/MASLD and Atrial Fibrillation (AF)

AF is the most common sustained arrhythmia, affecting approximately 60 million people globally, and increasing the risk of stroke four- to five-fold [57]. AF is also an established predictor of HFpEF with poor long-term outcomes [58], whilst it is also associated with an increased risk of MI and congestive heart failure (CHF) hospitalization [59]. Obesity, T2DM, dyslipidemia, and hypertension are shared risk factors for AF and NAFLD/MAFLD/MASLD [60]. Notably, the systemic, chronic low-grade inflammation and ectopic epicardial fat in steatosis/steatohepatitis appear to contribute to atrial and electrical myopathies, ultimately leading to AF [61,62]. Accordingly, growing clinical data have consistently demonstrated a significant association between NAFLD/MAFLD/MASLD and an increased risk of various cardiac arrhythmias, including AF, QT prolongation, ventricular tachyarrhythmias, and premature atrial and ventricular complexes [63,64,65,66,67,68,69,70]. The Framingham Heart Study, which involved 3744 individuals with a 10-year follow-up period, showed that high levels of aspartate and alanine transaminases were associated with an increased incidence of AF, as demonstrated by a HR of 1.12 (95% CI: 1.01 to 1.24) and 1.19 (95% CI: 1.07 to 1.32), respectively [65]. Additionally, a study by Targher et al., involving 400 individuals with T2DM who were followed for 10-years, found significantly higher AF incidence in the NAFLD group compared to the control group (OR: 4.49, 95% CI: 1.6 to 12.9) [66]. Another study by Targher et al., which further assessed the AF prevalence in 702 hospitalized patients with T2DM, also showed that the risk of AF was higher in individuals with NAFLD (OR: 3.04, 95% CI: 1.54 to 6.02), even after adjusting for other AF risk factors [67]. Similarly, a large study conducted in an Asian population (924,497 NAFLD and 5,309,434 non-NAFLD participants) followed up for 8 years showed that those with NAFLD have a 12% increased risk of AF compared to the controls [71]. Moreover, NAFLD was also identified as an independent predictor of AF in the Oulu Project Elucidating Risk of Atherosclerosis (OPERA) study, which had a longer follow-up period of 16.3 years and involved 958 individuals of whom 26% were diagnosed with NAFLD [72]. Although more than half of the participants of that study had hypertension, the results remained significant even after adjusting for all key AF risk factors (OR: 1.88, 95% CI: 1.03 to 3.45) [72]. However, analysis of data on 2122 individuals from the Framingham Heart Study who were diagnosed with NAFLD through computed tomography (CT) scans and were followed for 12 years showed that NAFLD was not significantly associated with AF after adjusting for factors such as age, smoking, diabetes, HF, and hypertension [73]. This may be attributed, at least in part, to insufficient statistical power to detect a potential association between AF and CT-diagnosed NAFLD, as both the prevalence and incidence of AF were notably low within the studied population [73]. Indeed, a larger study in the United States, which included 9108 hospitalized patients with NAFLD and 111,812 individuals without NAFLD, showed that the prevalence of AF was significantly higher in patients with NAFLD compared to those without (OR: 2.13, 95% CI: 1.93 to 2.34) [74]. Similarly, a study in South Korea, which included 232,979 individuals without a prior history of AF or structural heart disease, demonstrated that the AF risk was significantly higher in patients diagnosed with NAFLD, even after adjusting for factors such as elevated serum creatinine levels, HF, obesity, impaired fasting glucose, hypertension, and dyslipidemia (HR: 1.13, 95% CI: 1.03 to 1.24) [75]. Similar findings have been reported by a multicenter study by Pastori et al. among 1735 patients with non-valvular AF, of whom 732 (42.2%) were found to have NAFLD based on the FLI [76]. Additionally, this study over a median follow-up period of 18.7 months, also showed that patients with AF on anticoagulation therapy within the NAFLD group did not exhibit an increased risk of bleeding compared to those without NAFLD [76]. Such data merit further research attention, since it has been reported that four out of ten NASH patients were not receiving anticoagulation therapy despite meeting the corresponding criteria due to concerns regarding the risk of bleeding [74]. Although there have been limited studies on the safety and benefits of anticoagulation therapy in NAFLD with AF, the effectiveness in preventing strokes should be considered, whilst it is also important to note that the risk of bleeding is high in cases of decompensated liver disease [77,78].

Systematic review and meta-analysis data from five studies with a total of 238,129 individuals have also demonstrated a two-fold higher AF risk in patients with NAFLD compared to controls (RR: 2.06, 95% CI: 1.10 to 3.85) [79]. Another meta-analysis by Mantovani et al., with 364,919 individuals from nine studies investigating the incidence and prevalence of AF in patients with NAFLD, also revealed a higher risk of prevalent AF in patients with NAFLD compared to those without (OR: 2.07, 95% CI: 1.38 to 3.10) [80]. However, when data were stratified according to the type of included cohorts (cohorts with T2DM only vs. community-based or population-based cohorts), NAFLD presence was significantly associated with an increased 10-year risk of incident AF only in the cohort of outpatients with T2DM (HR: 4.96, 95% CI: 1.42 to 17.28) [80]. This appears to be in line with the role of older age and diabetes as risk factors for AF (e.g., individuals with diabetes exhibit a 40% increased AF risk compared to those without) [81,82]. It is also interesting to note that none of the studies in the meta-analysis by Mantovani et al. used 24-h Holter electrocardiogram (ECG) monitoring [80]; hence, these findings may not reflect the precise incidence of AF in patients with NAFLD, since cases of paroxysmal AF could have been missed [83]. Of note, recent systematic review and meta-analysis data on the incidence of AF among children and young adults aged ≤ 40 years (10,668,189 participants followed up for a median of 10.6 years) found that, even in this young population, the incident risk of AF was significantly higher in those with NAFLD (HR: 2.00, 95% CI: 1.12 to 3.57; *p* = 0.02) [48]. Although in this meta-analysis there was a variety of NAFLD diagnostic methods with variable sensitivity and specificity for moderate to severe steatosis, the reported results were also significant on subgroup analysis [48]. Interestingly, a study that involved only biopsy-confirmed NAFLD diagnosis in children and young adults (≤25 years) who were followed up for a median of 16.6 years revealed that the incident risk of AF was significantly higher in patients with NAFLD compared to controls [84]. A meta-analysis conducted by Zhou et al., with 14,213,289 participants and a median follow-up duration of 7.8 years, reported that the incidence of AF was significantly higher in the NAFLD group compared to the control (HR: 1.18, 95% CI: 1.12 to 1.23; *p* < 0.00001) [85]. However, two studies included in this meta-analysis used International Classification of Diseases (ICD) codes [86,87], thus introducing the possibility of misclassification bias. Indeed, on subgroup analysis of NAFLD diagnosis, there was no significant association between AF and NAFLD when diagnostic codes were used (HR: 1.00, 95% CI: 0.71 to 1.40; *p* = 0.99), while the AF and NAFLD association was significant when the FLI was used for the diagnosis of NAFLD (HR: 1.19, 95% CI: 1.13 to 1.25; *p* < 0.00001) [85]. Another meta-analysis by Alon et al. with a total of 8,115,545 individuals (34% with NAFLD) from various geographical areas (Europe, Asia, and North America) showed that the AF risk was significantly higher in patients with NAFLD compared to those without (OR: 1.27, 95% CI: 1.18 to 1.37) [46]. Moreover, a meta-analysis study by Bisaccia et al. (337,698 adults, including 84,511 with NAFLD; median follow-up of 24 years) also showed a significantly higher AF risk in those with NAFLD compared to the control group (OR: 1.68; 95% CI: 1.22 to 2.30) [88]. Although data on the association between AF and specifically MAFLD remain limited, a study by Lei et al. (54,832 participants; 33% with MAFLD) reported that those with MAFLD exhibited a high risk of developing AF (HR: 1.99, 95% CI: 1.39 to 2.83; *p* < 0.001) [89]. A recent meta-analysis by Mantovani et al. of 16 retrospective cohort studies (19,424,566 individuals of whom 2,487,792 had MASLD; median follow-up of 7.2 years) also showed that MASLD was associated with a significantly increased risk of incident AF (HR: 1.20, 95% CI: 1.10 to 1.32), independently of conventional cardio-metabolic risk factors [90]. Table 3 presents a summary of the results from key systematic reviews and meta-analyses on the association between NAFLD or MAFLD or MASLD and the development of AF [46,48,79,80,85,88,90,91].

## 5. NAFLD/MAFLD/MASLD and Heart Failure (HF)

**Figure 2:**
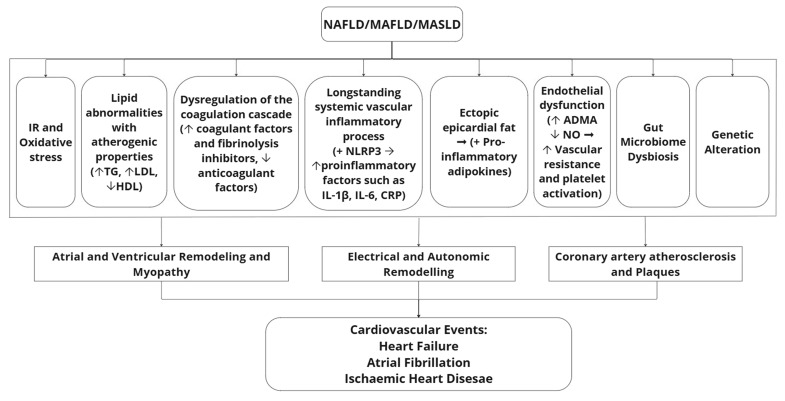
Simplified representation of key pathophysiological mechanisms implicated in the cardiovascular complications of non-alcoholic fatty liver disease (NAFLD), metabolic dysfunction-associated fatty liver disease (MAFLD), and metabolic dysfunction-associated steatotic liver disease (MASLD). Here, regarding the general pathophysiology aspects of the disease, the terms NAFLD, MAFLD and MASLD are applied interchangeably and depicted as NAFLD/MAFLD/MASLD. Abbreviations: ADMA: Asymmetric dimethyl arginine; CRP: C-reactive protein; HDL: High-density lipoprotein; IL-1β: Interleukin-1β; IL-6: Interleukin-6; IR: Insulin resistance; LDL: Low-density lipoprotein; NO: Nitric oxide; NLRP3: Nucleotide-binding domain, leucine-rich-containing family, pyrin domain-containing protein 3 patatin-like phospholipase domain-containing protein 3; TG: Triglycerides. The symbols “+”, “↓”, and “↑” are used to indicate activation, decrease, and increase, respectively. The symbol “→” is used to indicate progression.

HF is a rapidly growing public health issue affecting 64.3 million people worldwide [92] and resulting in high mortality and morbidity with recurrent hospitalisations and reduced quality of life [93]. As with other cardiac diseases, HF shares key common risk factors with NAFLD/MAFLD/MASLD, such as obesity, T2DM, dyslipidemia, and hypertension [94]. However, regardless of the presence of such metabolic syndrome-related diseases, compelling evidence further indicates a significant independent association of NAFLD with the development of LVH and diastolic dysfunction [70], which has been observed not only in adult patients with NAFLD, but also in children [95]. As illustrated in Figure 2, NAFLD/MAFLD/MASLD appears to be implicated in atrial, ventricular, and electrical remodeling and myopathies through complex pathophysiological mechanisms [61,62,96].

As such, a prospective population-based cohort study of 1827 individuals with CT-diagnosed NAFLD followed for five years showed that NAFLD is associated with subclinical LV remodeling and hypertrophy, as well as impaired myocardial strain, independently of other HF risk factors [97]. Furthermore, a small study in 65 individuals without known CVD of whom 14 had biopsy-proven NASH also showed that NASH was associated with myocardial structure alterations, whilst it was also inversely correlated with indices of LV diastolic function [98]. In another study from Korea with 20,821 individuals (30% with NAFLD), the NAFLD group had abnormal LV relaxation on echocardiography which correlated with NAFLD severity, as well as increased relative wall thickness compared to the non-NAFLD group, suggesting significant LV structural and functional alteration [99]. Echocardiography findings of a higher LV mass and end-diastolic volume, as well as increased LV relative wall thickness, in patients with CT-diagnosed NAFLD compared to controls were also reported by the multicenter, community-based, Coronary Artery Risk Development in Young Adults (CARDIA) study in a cohort of 2713 young adults (271 with NAFLD) as part of the 25-year follow-up examination [100]. Additionally, a study in 308 participants (38% with NAFLD) revealed a significant correlation between hepatic steatosis and fibrosis as assessed by transient liver elastography with LV diastolic dysfunction as evaluated by 18F-fluorodeoxyglucose-positron emission tomography and echocardiography [101]. An association of hepatic steatosis with an increased LV mass index, LV wall thickness, and LV filling pressure, as well as increased mitral peak velocity, was also documented in a cross-sectional study in 2356 adults (384 with hepatic steatosis) who underwent echocardiography and hepatic CT scans [102]. In that study, hepatic steatosis was also inversely correlated with global systolic longitudinal strain and diastolic annular velocity, indicating multiple subclinical systolic and diastolic cardiac dysfunctions in such patients [102]. Another study in 228 individuals (75% with MAFLD) also showed that LV diastolic dysfunction was significantly more prevalent in the MAFLD group compared to the controls (60.8% vs. 24.6%, respectively, *p* < 0.001) [103]. Interestingly, a study involving 147 patients with biopsy-proven NAFLD revealed that NAFLD was associated with increased epicardial fat accumulation, which correlated with fibrosis severity [104]. That study further identified significant alterations in cardiac structure, such as increased posterior wall thickness, increased relative wall thickness, and increased left atrium volume, which also correlated with fibrosis severity, whilst a negative correlation with LV ejection fraction was also documented [104]. A significant association between the prevalence of LV diastolic dysfunction and the NAFLD fibrosis grade has also been identified in a large study including 1310 patients with NAFLD diagnosed by ultrasonography and 1990 controls (30.4%, 35.2%, and 57.4% prevalence among those without NAFLD, with NAFLD without advanced fibrosis, and with NAFLD with advanced fibrosis, respectively, *p* < 0.001) [105]. That study also found that the increased risk for LV diastolic dysfunction according to the NAFLD fibrosis grade was more pronounced among those with a BMI less than 25 kg/m^2^ compared to those with obesity, independent of other risk factors [105]. Furthermore, meta-analysis data from 16 studies (total of 26,365 participants; 67% with NAFLD) also support the observed association between NAFLD and subclinical cardiac structural alterations, with the NAFLD group exhibiting higher LV mass, LV end-diastolic volume, and left atrium diameter, as well as increased posterior wall and septal thickness, compared to the non-NAFLD group, thus indicating subclinical LV diastolic dysfunction in NAFLD [106]. Another meta-analysis of 41 studies (total of 33,891 patients) found that NAFLD was associated with impairment of both systolic and diastolic cardiac function, as well as with changes in cardiac structure (increased LV mass and epicardial adipose thickness) [107]. Increased NAFLD severity was associated with worse diastolic [e.g., decreased early to late diastolic transmitral flow velocity as assessed echocardiographically by the peak E wave (E) to peak A (A) wave (EA) ratio], but not with systolic indices [107]. Diastolic echocardiographic parameters, such as the E to early diastolic mitral annular tissue velocity (E/e’), are markers of LV diastolic dysfunction, with increased LV filling pressures reflecting an increased E/e’ ratio [108]. A higher E/e’ ratio in patients with NAFLD compared to controls [standardized mean difference (SMD) between the two groups of 1.02; 95% CI: 0.43 to 1.61] has been documented in a recent systematic review and meta-analysis of 21 studies with a total of 35,013 participants (30% with NAFLD) [109]. Furthermore, this meta-analysis also showed structural cardiac changes in the NAFLD group compared to controls, including increases in both the LV mass index (SMD: 0.89, 95% CI: 0.31 to 1.47) and the left atrium volume index (SMD: 0.87, 95% CI: 0.38 to 1.37), suggesting that patients with NAFLD are at a higher risk of LV diastolic dysfunction [109].

The aforementioned adverse cardiac structural changes can progressively contribute to the development and progression of new-onset HF [110]. A community-based cohort study in 3544 Framingham Study participants followed for a mean duration of 23 years found that a mild increase in GGT levels was associated with a higher incidence of HF, independently of HF risk factors [111]. These findings were consistent with those from both the British Regional Heart Study (3494 participants followed for a mean period of 9 years), and the FINRISK cohort study (38,079 participants followed for a mean period of 14.5 years), which also showed that an increase in GGT levels was significantly associated with a higher incidence of HF in these community-based cohorts [112,113]. Another study in the United States, which included 3869 patients with NAFLD and 15,209 controls who were followed over a median duration of 7 years, identified a higher incidence of developing HF in the NAFLD group (HR: 1.47, 95% CI: 1.27 to 1.70) [114]. Similarly, UK Biobank data from 196,198 individuals without baseline HF or other CVD who were followed for a median of 8 years revealed that those with NAFLD, based on a high FLI, had a significantly higher incidence of developing HF (HR: 1.74, 95% CI: 1.63 to 1.86) [115]. Moreover, a cohort study including 8,962,813 healthy Koreans followed for a median of 10 years also showed that individuals with a high FLI (FLI > 30) had a significantly higher risk for developing new-onset HF (HR: 1.61, 95% CI: 1.55 to 1.67), independently of other established HF risk factors [116]. A higher risk of HF incidence has also been noted in biopsy-proven NAFLD, as shown by a nationwide Swedish cohort study (10,422 adults with biopsy-proven NAFLD without baseline CVD followed for a median of 13.6 years) [42]. This significant association between a higher risk of developing HF and NAFLD (HR: 1.75, 95% CI: 1.63 to 1.87) was independent of known HF risk factors and correlated with NAFLD severity, since the HF incidence was higher in patients with cirrhosis (HR: 2.83, 95% CI: 2.08 to 3.85) compared to those with hepatic fibrosis without cirrhosis (HR: 2.04, 95% CI: 1.66 to 2.51) [42]. Another cohort study (870,535 participants without a prior history of CVD, of whom 27,919 had NAFLD; mean follow-up of 14.3 years) also showed that NAFLD had a significant independent association with an increased HF risk of new-onset HF (adjusted HR: 1.23, 95% CI: 1.18 to 1.29) [117]. The risk of HFpEF in this study was significantly higher compared to that of HF with reduced ejection fraction (HFrEF), with HR of 1.24 (95% CI: 1.14 to 1.34) and 1.09 (95% CI: 0.98 to 1.20), respectively [117]. A small study with 181 participants (27% with NAFLD) also showed a two-fold higher risk of HFpEF in patients with NAFLD compared to those without, with this risk being higher in the patients with liver fibrosis and cirrhosis [118]. A significant association between NAFLD and the risk of developing new-onset HF (HR: 1.34, 95% CI: 1.28 to 1.39; *p* < 0.001) during a 10-year follow-up period was also shown in a retrospective analysis of 173,966 adult outpatients in Germany (50% with NAFLD) [119]. Similar findings have been reported for MAFLD, with a large study which followed individuals without baseline HF for 14 years showing that patients with MAFLD (N = 30,755) had an increased risk of HF (HR: 1.40, 95% CI: 1.30 to 1.50) compared to those without (N = 67,930) [120]. Moreover, a significantly increased HF incidence in patients with MASLD compared to those without (HR: 1.38, 95% CI: 1.35 to 1.41) has been documented in a study with 8,808,494 individuals without baseline CVD (27.5% with MASLD) and a median follow-up of 12 years [121]. A meta-analysis by Li et al., with a total of 10,979,967 participants (22.2% with NAFLD), also showed an increased HF risk in the NAFLD group (HR: 1.36, 95% CI: 1.16 to 1.58), even after adjustment for several confounding risk factors [122]. In addition, a systematic review and meta-analysis study of 11 cohort studies (11,242,231 individuals from the United States, Europe, and Asia; 26.2% with NAFLD; median follow-up of 10 years) showed that patients with NAFLD had an increased risk of new-onset HF (HR: 1.50, 95% CI: 1.34 to 1.67), independently of known HF risk factors [123].

It is important to also note that adverse cardiac structural remodelling significantly impacts not only on the onset, but also on the progression of HF [110]. Accordingly, there are data showing that patients with FLI ≥ 60 exhibit an increased HF incidence (HR: 1.30, 95% CI: 1.24 to 1.36), increased HF hospitalization (HR: 1.54, 95% CI: 1.44 to 1.66), and increased cardiovascular mortality (HR: 1.41, 95% CI: 1.22 to 1.63) [124]. Moreover, a study in 264 older patients with NAFLD (mean age: 83 ± 9 years) who were followed on average for nearly two years showed an association of NAFLD with an increased risk of hospital admission and post-discharged HF mortality (HR: 1.82, 95% CI: 1.22 to 2.81; *p* < 0.001) even after adjustment for potential confounders [125]. Progression and worse prognosis of HF in patients with NAFLD was also evident in a recent systematic review and meta-analysis (12,374 patients with HF; median follow-up of 2.5 years) which showed that those with NAFLD had a significantly higher risk of primary adverse outcomes (HR: 1.61, 95% CI: 1.25 to 2.07), all-cause mortality (HR: 1.66, 95% CI: 1.39 to 1.98), and HF hospitalization or re-hospitalization (HR: 1.71, 95% CI: 1.03 to 2.86) compared to those without [126]. A summary of these findings from key systematic reviews and meta-analyses on the association between NAFLD and HF events is presented in Table 4 [46,122,123,127,128].

## 6. Pathophysiological Nexus Between NAFLD/MAFLD/MASLD and CVD

Given the aforementioned growing body of data on the association between NAFLD/MAFLD/MASLD and CVD, it is important to briefly highlight key underlying mechanisms which are considered to play a mediating pathophysiologic role (Figure 2). The hallmark of the pathophysiology of NAFLD/MAFLD/MASLD is the accumulation of fat in the liver (steatosis), which is considered to represent the initial pathophysiological insult [129]. Following this and particularly in the context of obesity, the liver is frequently exposed to high circulating levels of pro-inflammatory adipokines and cytokines secreted from the adipose tissue, as well as mitochondrial dysfunction, endoplasmic reticulum (ER) stress, and oxidative stress in hepatocytes [130,131,132]. All these promote hepatic inflammation (steatohepatitis; NASH or MASH) and hepatocellular injury and may induce progression from simple steatosis [129]. This progression is considered a key factor for the subsequent development of hepatic and extrahepatic complications in the context of NAFLD/MAFLD/MASLD, with steatohepatitis being already present in more than a quarter of adults at the time of diagnosis [10,133].

The complete pathophysiological nexus between NAFLD/MAFLD/MASLD and CVD is still not fully understood, since complex and multifactorial underlying mechanisms are implicated [10]. These appear to primarily involve obesity-related chronic low-grade inflammation and insulin resistance, as well as atherogenic lipid abnormalities, ectopic epicardial fat accumulation, gut microbiota dysbiosis, and dysregulation in the balance between pro- and anti-coagulant factors [10,28,134,135,136,137,138,139,140] (Figure 2). Genetic factors and endothelial dysfunction are also implicated in the pathophysiological links between NAFLD/MAFLD/MASLD and CVD [28,141,142,143], as outlined in Figure 2. Collectively, all these factors are considered to play a role in a ‘multi-hit’ pathophysiologic model which characterizes chronic hepatic steatosis and particularly steatohepatitis [144,145,146,147]. In this context, metabolic dysfunction and dyslipidemia appear to act as primary disease triggers, with insulin resistance precipitating higher free fatty acid (FFA) accumulation within the liver [148,149]. This may result in lipotoxicity, characterized by the degradation of hepatocyte membranes and subsequent release of pro-inflammatory mediators, which in turn exacerbates insulin resistance, thus creating a vicious pathophysiological cycle [150,151,152]. Consequently, the liver initiates a ductular reaction as a compensatory mechanism aimed at local tissue repair, which, however, may contribute to the progression of hepatic fibrosis when this reaction persists [153]. The dysregulation of lipid metabolism leads to high levels of low-density lipoprotein (LDL) cholesterol and triglycerides, with low levels of HDL cholesterol, thus promoting atherogenic dyslipidemia [154,155]. For example, it has been shown that angiopoietin-like protein 8 (ANGPTL8) is involved in hypertriglyceridemia through the inhibition of the lipoprotein lipase enzyme [156], which is responsible for triglyceride breakdown [157]. Subsequently, this atherogenic dyslipidemia triggers the activation of toll-like receptors (TLR) 2 and 4, penetrating the vascular wall and resulting in the activation of the nucleotide-binding domain, leucine-rich–containing family, pyrin domain-containing protein 3 (NLRP3) inflammasome [137,158]. The NLRP3 induces ongoing low-grade, chronic inflammation by inducing the release of pro-inflammatory cytokines, such as interleukin (IL)-1β, IL-6, and CRP, thereby promoting atherosclerotic plaque formation [144]. The Multi-Ethnic Study of Atherosclerosis (MESA), involving 3876 participants from the general population (668 with NAFLD), showed that IL-6 was independently associated with subclinical atherosclerosis, as indicated by a high CAC score, suggesting that IL-6 is not only linked to the presence of subclinical atherosclerosis, but also to its severity [159]. Mendelian randomization studies have also found that impairment of the IL-6 pathway functionality results in decreased long-term vascular events [160]. Additionally, CRP has been identified as a prognostic biomarker, independently predicting CVD mortality in individuals with MAFLD [161]. Overall, polygenic risk scores in combination with novel biomarkers (e.g., through metabolomics proteomics and transcriptomics), particularly focusing on pro-inflammatory and pro-atherogenic processes, appear to be screening and diagnostic tools for the prompt and effective monitoring of the broader cardiovascular–liver–metabolic health [162]. The systemic, chronic inflammation in NAFLD/MAFLD/MASLD may also cause atrial myopathy, and contribute to AF and ventricular myopathy, thus causing ventricular remodeling and ultimately HFpEF [61,96]. This is supported by meta-analysis data which revealed a significant association between elevated levels of pro-inflammatory biomarkers and an increased incidence of AF [163]. Additionally, this pro-inflammatory process may further favor the accumulation of ectopic epicardial fat surrounding the atria, ventricles, or coronary arteries [164,165]. This epicardial fat serves as an additional local source of pro-inflammatory adipokines, such as leptin, which contribute to myocardial myopathy and fibrosis, thereby playing a significant role in the pathogenesis of AF and HFpEF [166,167]. Furthermore, the presence of ectopic epicardial fat contributes to electrical remodeling, leading to a reduction in the effective refractory period [62]. Over time, this alteration promotes the development of chaotic electrical patterns, ultimately resulting in AF [62]. As such, ectopic epicardial fat has been identified as an independent predictor for the development of AF [168]. Epicardial fat adjacent to the coronary arteries has also been shown to release additional adipokines, such as resistin, which may contribute to the development of CAD [169]. Overall, meta-analysis data indicate that epicardial fat in NAFLD may be an independent risk factor for CAD, cardiac arrhythmias, and CHF [170].

Moreover, the gut microbiome is also emerging as an additional pathophysiologic factor in patients with steatosis/steatohepatitis and appears to also play a role in the pathophysiology of plaque formation in such patients (Figure 2) [171]. Indeed, alterations in the gut microbiota appear to disrupt cholesterol and triglyceride metabolism, thereby contributing to dyslipidemia and atherogenesis [141,172]. For example, gut microbiome alterations impact on choline and carnitine metabolism, leading to elevated levels of trimethylamine oxide (TMAO), which has been linked to the formation of atherosclerotic plaques [173]. Additionally, TMAO serves as a prognostic indicator for both short- and long-term cardiovascular complications in patients with ACS [174]. Genetic polymorphisms in patatin-like phospholipase domain-containing protein 3 (PNPLA3), transmembrane 6 superfamily member 2 (TM6SF2), membrane-bound O-acyltransferase domain-containing 7 (MBOAT7), and glucokinase regulatory protein (GCKR) are also considered to play a significant role in steatohepatitis, fibrosis, and hepatic carcinogenesis [175,176]. However, these genetic variants exhibit diverse effects on CVD risk, as certain variants exhibit cardio-protective effects (e.g., the E167K variant of TM6SF2 and the I148M variant of PNPLA3) [177,178,179], while others increase the cardio-metabolic risk (e.g., the rs738409 variant of PNPLA3) [180]. As such, evidence suggests that the rs738409 PNPLA3 variant predicts CAD and could serve as a relevant diagnostic biomarker [180]. A study in patients with biopsy-proven NAFLD showed that the PNPLA3 GG genotype exhibits a significant association with higher severity of carotid atherosclerosis in younger patients with NAFLD [181]. Notably, Zhong et al. also identified eight co-upregulated and 31 co-downregulated genes between NAFLD and AF [182]. Genes such as AMOT, PDE11A, TYMS, TMEM98, and PTGS2 demonstrated substantial diagnostic potential for identifying NAFLD patients at risk of AF [182]. Moreover, they suggest that mitochondrial disturbances may underpin the systemic inflammation in NAFLD, which possibly exacerbates AF [182]. Furthermore, dysregulation of the coagulation cascade is evidenced in patients with NAFLD, resulting in a hypercoagulable state characterized by increased levels of coagulation and fibrinolysis inhibitors, as well as decreased levels of anticoagulant factors (e.g., protein C and S), thus promoting athero-thrombosis [183,184,185]. In addition, endothelial dysfunction, an early process in atherosclerosis, appears to also play a key role in the pathophysiological links between steatotic liver disease and CVD [157]. This dysfunction appears associated with impaired regulation of homocysteine and asymmetric dimethyl arginine (ADMA) metabolism in the liver, leading to their local accumulation [146,186]. Consequently, elevated homocysteine and ADMA levels reduce nitric oxide (NO) production, resulting in increased vascular resistance and platelet activation [145,187,188]. Since endothelial dysfunction appears to contribute to the underlying pathophysiology of atherogenesis in NAFLD/MAFLD/MASLD, targeting endothelial cells could be an additional strategy in developing novel treatments for CVD in patients with steatosis/steatohepatitis [143].

## 7. Future Perspectives Related to the Pathophysiology, Diagnosis, and Management of MASLD

As aforementioned, significant progress has been made during the past few decades in elucidating the pathophysiology of MASLD, with both translational and clinical research studies establishing strong links between this highly prevalent liver disease and CVD [189,190,191,192,193]. Based on this increasing body of evidence which links MASLD and CVD, recent recommendations and the clinical practice guidelines by the European Association for the Study of the Liver (EASL), the European Association for the Study of Diabetes (EASD), the European Association for the Study of Obesity (EASO), and the American Diabetes Association (ADA) recognise MASLD as a key modifiable CVD risk factor and recommend systematic cardiovascular assessment and integrated management to mitigate adverse cardiovascular–liver–metabolic health (CLMH) outcomes [189,190,191]. Indeed, consistent evidence increasingly shows that particularly MASH and advanced liver fibrosis are associated not only with progression to cirrhosis, but also with elevated CVD risk [192,193]. Meta-analysis data have also shown that non-invasive fibrosis scores, such as the NFS and FIB-4, can predict CVD events and mortality, even after adjusting for age, BMI, T2DM, and hypertension [194]. Moreover, data from general population cohorts also indicate that higher hepatic fibrosis scores are associated with CVD events [44], independently of traditional cardio-metabolic risk factors, suggesting that hepatic fibrosis itself may be considered as a cardio-metabolic risk factor. Accordingly, considering advanced hepatic fibrosis as an additional independent CVD risk factor could potentially refine cardio-metabolic risk stratification models and help to better inform the relevant monitoring and management strategies. In this context, non-invasive evaluation of liver fibrosis is particularly important in patients with multiple cardio-metabolic risk factors, such as obesity, T2DM, hypertension, and dyslipidaemia. As such, a number of serum-based scoring systems have been developed—collectively termed by the American Association for the Study of Liver Diseases (AASLD) as non-invasive liver disease assessment(s) (NILDAs) [195]—to estimate steatosis and fibrosis by integrating routinely measured clinical and biochemical parameters, including the NFS, FIB-4 and the Enhanced Liver Fibrosis (ELF) test [195,196,197,198]. These NILDAs can be particularly valuable to better stratify the risk of adverse CLMH outcomes at the population level and identify high-risk individuals who may benefit from further evaluation for the detection of advanced hepatic inflammation and fibrosis [195,196,197,198], thus facilitating timely interventions to prevent both hepatic and CVD complications.

**Figure 3:**
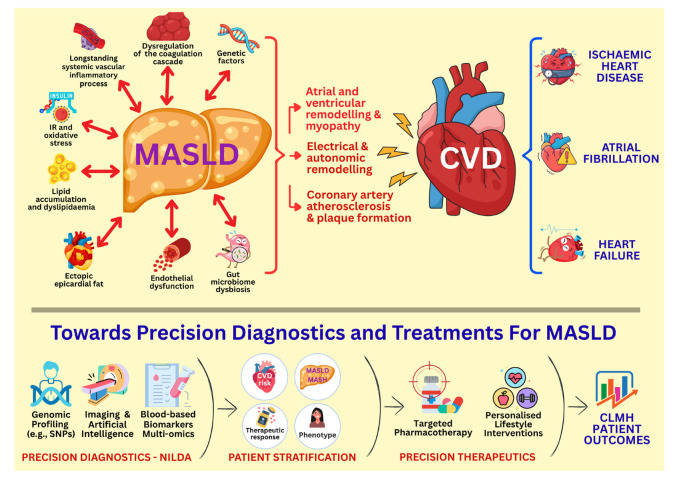
Clarifying the mechanisms which mediate the pathophysiology of metabolic dysfunction-associated steatotic liver disease (MASLD) and its links to cardiovascular disease (CVD) is expected to improve precision medicine approaches for MASLD diagnostics [e.g., blood-based and imaging-based non-invasive liver disease assessment(s) (NILDAs) together with artificial intelligence and multi-omics technologies], as well as for both pharmacological [e.g., glucagon-like peptide-1 receptor agonists (GLP-1RAs); GLP-1 and glucose-dependent insulinotropic polypeptide (GIP) co-agonists; triple hormone agonists of GLP-1, GIP, and glucagon receptors; sodium glucose cotransporter-2 inhibitors (SGLT2i); fibro-blast growth factor 21 analogues; proliferator-activated receptor (PPAR) agonists; farnesoid X receptor (FXR) agonists; and resmetirom, a selective thyroid hormone receptor beta (THR-β) agonist] and non-pharmacological (e.g., personalised dietary and exercise recommendations) interventions for patients with MASLD who are at high risk for adverse cardiovascular–liver–metabolic health (CLMH) outcomes.

It becomes evident that elucidating the complex pathophysiological interplay between MASLD and CVD, particularly regarding the role of novel mediators/biomarkers and the associations of MASLD with atherosclerotic disease and adverse electrical and structural processes of the heart, can further advance precision diagnostics and effective management strategies for this chronic liver condition (Figure 3). To address such knowledge gaps, further research focus is currently placed on the precise mechanisms mediating these complex interactions which impact on CLMH outcomes and remain incompletely understood, thus representing a critical area for further investigation. For example, the exact pathways through which hepatic lipid accumulation contributes to adverse CVD outcomes are not fully elucidated, particularly in lean patients with MASLD who lack key cardio-metabolic risk factors, such as obesity, and are estimated to be at least 7% of the individuals with MASLD [199,200]. Furthermore, despite the considerable recent advances, the identification and validation of novel reliable biomarkers for MASLD diagnosis, stratification, and monitoring remains challenging [195,196]. Both blood-based and imaging-based NILDAs have demonstrated potential for evaluating disease activity and fibrosis [195,201], but their diagnostic performance exhibits heterogeneity across clinical settings and patient populations. Thus, gaps still exist regarding NILDAs which are capable of effectively diagnosing MASLD and/or dynamically tracking MASLD/MASH progression and/or therapeutic responses, highlighting an unmet need for further research in this field. The development of such novel biomarkers with the integration of artificial intelligence and multi-omics technologies is expected not only to improve early detection, diagnostic accuracy, risk stratification and dynamic monitoring for MASLD, but also to enable more personalized/tailored therapeutic approaches and treatment planning for these patients (Figure 3) [162,195,201,202]. For such precision medicine approaches, more research is also required for the new pharmacologic agents against MASLD which target obesity and/or hyperglycaemia [e.g., glucagon-like peptide-1 receptor agonists (GLP-1RAs); GLP-1 and glucose-dependent insulinotropic polypeptide (GIP) co-agonists; triple hormone agonists of GLP-1, GIP, and glucagon receptors; and sodium glucose cotransporter-2 inhibitors (SGLT2i)] or other pathways implicated in MASLD/MASH [e.g., fibroblast growth factor 21 analogues; proliferator-activated receptor (PPAR) agonists; farnesoid X receptor (FXR) agonists; and resmetirom, a selective thyroid hormone receptor beta (THR-β) agonist, which is the first FDA-approved drug for MASH] [203,204,205,206,207,208,209,210]. Indeed, such new—approved and under development—pharmacological treatments targeting MASLD/MASH require further clinical research, particularly regarding their long-term efficacy, safety profile and CLMH benefits, whilst effective direct anti-fibrotic agents are still missing [203,204,205,206,207,208,209,210]. Finally, it should also be highlighted that lifestyle modification remains a cornerstone for the effective long-term management of patients with MASLD/MASH alongside any pharmacological treatment, and, hence, research is also needed to optimise the benefits of these non-pharmacological interventions. Accordingly, there are knowledge gaps regarding the optimal and personalised dietary approaches for patients with MASLD/MASH (e.g., the long-term effects of the Mediterranean diet, caloric restriction, intermittent fasting, low-carbohydrate and ketogenic diets, as well as the therapeutic potential of prebiotics, probiotics, synbiotics, and other gut microbiota-based interventions) which need to be further investigated [171,211,212,213,214,215]. Optimising the long-term effects of these dietary interventions, together with personalised recommendations for physical activity and exercise [190,211,212,213,214,215,216], may further benefit long-term CLMH outcomes in the context of a holistic and individualised therapeutic approach for patients with MASLD/MASH (Figure 3).

## 8. Conclusions

Consistent evidence supports a significant association between metabolic-related steatosis/steatohepatitis and CVD, including not only CAD, but also AF and HF. This association appears to be independent of other known CVD risk factors (despite the synergy and significant overlap between all these cardio-metabolic diseases), thus highlighting this highly prevalent hepatic disease as a key cardio-metabolic risk factor, which merits early diagnosis and treatment in order to promptly reduce the related CVD risk. Indeed, development of NAFLD/MAFLD/MASLD-related cardiovascular morbidity appears to progressively increase with the severity of the underlying hepatic pathology, particularly with the progression to steatohepatitis and fibrosis. The evidence presented in this review highlights the links between NAFLD/MAFLD/MASLD and CVD through cardiac structural, electrical, and functional alterations which can progress to clinical manifestations of CAD, AF, and new-onset HF. Recognising these links in routine clinical practice further underscores the importance of early detection and multi-disciplinary management of metabolic-related steatosis/steatohepatitis (e.g., by hepatologists, endocrinologists and cardiologists) in order to prevent both disease progression and CVD complications. In this context, further clinical and translational research is also warranted to explore novel biomarkers and pharmacotherapies for MASLD which can be utilized, respectively, for the early diagnosis/monitoring and effective treatment of cardio-metabolic complications in these patients.

## Figures and Tables

**Table 1:** Key systematic reviews and meta-analyses on the associations between subclinical atherosclerosis and non-alcoholic fatty liver disease (NAFLD), or metabolic dysfunction-associated steatotic liver disease (MASLD).

Author, Year, [Reference]	Meta-Analysis Characteristics	Subclinical Atherosclerosis Outcome	OR or MD (95% CI)
Zhou et al., 2018, [36]	Total Number of Studies: 26 Total Population: 85,395 NAFLD Population: 29,493	Increased CIMT or plaques	OR: 1.74 (1.47 to 2.06)
		Increased arterial wall stiffness	OR: 1.56 (1.24 to 1.96)
		Increased CAC score	OR: 1.40 (1.22 to 1.60)
		Reduced endothelial function	OR: 3.73 (0.99 to 14.1)
Wong et al., 2021, [37]	Total Number of Studies: 64 Total Population: 172,385 NAFLD Population: 67,404	Increased CIMT	OR: 2.00 (1.56 to 2.56)
		Increased CAC score	OR: 1.21 (1.12 to 1.32)
		CAC score progression	OR: 1.26 (1.04 to 1.52)
Koulaouzidis et al., 2021, [38]	Total Number of Studies: 5 NAFLD Population: 10,060	CAC score progression	OR: 1.50 (1.34 to 1.68)
Toh et al., 2022, [39]	Total Number of Studies: 24 NAFLD Population: 62,623	Coronary artery stenosis or plaques, or increased CAC score	OR: 1.22 (1.13 to 1.31)
Jamalinia et al., 2023, [30]	Total Number of Studies: 12 NAFLD Population: 4725	Increased CIMT, arterial wall stiffness, or CAC score	OR: 2.18 (1.62 to 2.93)
Abosheaishaa et al., 2024, [40]	Total Number of Studies: 59 Total Population: 37,773 NAFLD Population: 13,861	Increased CIMT	MD: 0.10 (0.09 to 0.12)
De Filippo et al., 2024, [41]	Total Number of Studies: 12 MASLD Population: 41,243	Increased CAC score	OR: 2.26 (1.55 to 3.23)
Mladenova et al., 2025, [35] *	Total Number of Studies: 83 Total Population: 54,064 NAFLD Population: 21,458	Increased CIMT	MD: 0.10 (0.09 to 0.11)
Mladenova et al., 2025, [35] *	Total Number of Studies: 12 Total Population: 5186 NAFLD Population: 2646	Carotid plaque formation	OR: 2.08 (1.52 to 2.86)

*: The study by Mladenova et al. (2025) [35] presents meta-analyses for both the association between NAFLD and CIMT and the association between NAFLD and carotid stenosis with carotid plaque formation as the endpoint. Abbreviations: CAC: Coronary Artery Calcium; CI: Confidence Interval; CIMT: Carotid Intima Media Thickness; MASLD: Metabolic Dysfunction-Associated Steatotic Liver Disease; MD: Mean Difference; NAFLD: Non-alcoholic Fatty Liver Disease; and OR: Odds Ratio.

**Table 2:** Key systematic reviews and meta-analyses on the association between clinical cardiovascular events and non-alcoholic fatty liver disease (NAFLD), or metabolic dysfunction-associated fatty liver disease (MAFLD).

Author, Year, [Reference]	Meta-Analysis Characteristics	Clinical Cardiovascular Outcome	OR or HR or RR (95% CI)
Targher et al., 2016, [43]	Total Number of Studies: 16 Total Population: 34,043 NAFLD Population: 12,361	Fatal and nonfatal CVD events	HR: 1.64 (1.26 to 2.13)
		Nonfatal CVD events	HR: 2.52 (1.52 to 4.18)
Wu et al., 2016, [54]	NAFLD Total Number of Studies: 64 Total Population: 164,494	CAD events	HR: 2.31 (1.46 to 3.65)
Haddad et al., 2017, [56]	Total Number of Studies: 6 Total Population: 25,837 NAFLD Population: 5953	CAD events	RR: 2.26 (1.04 to 4.92)
		Stroke events	RR: 2.09 (1.46 to 2.98)
Mantovani et al., 2021, [55]	Total Number of Studies: 36 Total Population: 5,800,000 NAFLD Population: 335,132	Any fatal or nonfatal CVD events	HR: 1.45 (1.31 to 1.61)
		Nonfatal CVD events	HR: 1.40 (1.20 to 1.64)
Toh et al., 2022, [39]	Total Number of Studies: 14 NAFLD Population: 67,070	CAD events	OR: 2.18 (1.69 to 2.81)
Prasad et al., 2023, [45]	NAFLD Total Number of Studies: 36 Total Population: 7,068,007	Fatal and nonfatal CVD events	HR: 1.41 (1.13 to 1.76)
		Nonfatal CVD events	HR: 1.57 (1.33 to 1.85)
Abosheaishaa et al., 2024, [53]	NAFLD Total Number of Studies: 32 Total Population: 5,610,990	Angina events	RR: 1.45 (1.17 to 1.79)
		CAD events	RR: 1.21 (1.07 to 1.38)
Mladenova et al., 2025, [35]	Total Number of Studies: 25 Total Population: 5,509,423 NAFLD Population: 593,635	ACS events	OR: 1.95 (1.49 to 2.55)
Jamalinia et al., 2025, [49]	MAFLD Total Number of Studies: 36 Total Population: 18,524,532	Fatal and nonfatal CVD events in males	HR: 1.37 (1.27 to 1.48)
		Fatal and nonfatal CVD events in females	HR: 1.59 (1.44 to 1.75)

Abbreviations: ACS: Acute Coronary Syndrome; CAD: Coronary Artery Disease; CI: Confidence Interval; CVD: Cardiovascular Disease; HR: Hazard Ratio; MAFLD: Metabolic Dysfunction-Associated Fatty Liver Disease; NAFLD: Non-alcoholic Fatty Liver Disease; OR: Odds Ratio; and RR: Relative Risk. Nonfatal CVD Events: including CAD, Myocardial Infarction, Coronary Revascularization Interventions, and Stroke.

**Table 3:** Key systematic reviews and meta-analyses on the association between atrial fibrillation (AF) and non-alcoholic fatty liver disease (NAFLD), metabolic dysfunction-associated fatty liver disease (MAFLD), and metabolic dysfunction-associated steatotic liver disease (MASLD).

Author, Year, [Reference]	Meta-Analysis Characteristics	Median Follow-Up	Clinical Cardiovascular Outcome	OR or HR or RR (95% CI)
Wijarnpreecha et al., 2017, [79]	Total Number of Studies: 5 Total Population: 238,129 NAFLD Population: 36,707	-	Incidence of AF	HR: 2.06 (1.10 to 3.85)
Mantovani et al., 2019, [80]	Total Number of Studies: 9 Total Population: 364,919 NAFLD Population: 156,915	-	Incidence of AF Prevalence of AF	HR: 1.16 (0.91 to 1.48) OR: 2.07 (1.38 to 3.10)
Cai et al., 2020, [91]	Total Number of Studies: 6 Total Population: 614,673 NAFLD Population: 245,869	10 years	Incidence of AF	RR: 1.19 (1.04 to 1.31)
Alon et al., 2022, [46]	Total Number of Studies: 7 Total Population: 8,115,545 NAFLD Population: 2,766,117	4 years	Incidence of AF	OR: 1.27 (1.18 to 1.37)
Bisaccia et al., 2023, [88]	Total Number of Studies: 4 Total Population: 337,698 NAFLD Population: 84,511	24 years	Incidence of AF	OR: 1.68 (1.22 to 2.30)
Zhou et al., 2023, [85]	Total Number of Studies: 12 NAFLD/MAFLD Population: 14,213,289	7.8 years	Incidence of AF	HR: 1.18 (1.12 to 1.23)
Liao et al., 2024, [48]	Total Number of Studies: 4 Total Population: 10,668,189 NAFLD Population: 1,068,246	10 years	Incidence of AF	HR: 2.00 (1.12 to 3.57)
Mantovani et al., 2025, [90]	Total Number of Studies: 16 Total Population: 19,424,566 MASLD Population: 2,487,792	7.2 years	Incidence of AF	HR: 1.20 (1.10 to 1.32)

Abbreviations: AF: Atrial Fibrillation; CI: Confidence Interval; HR: Hazard Ratio; MAFLD: Metabolic Dysfunction-Associated Fatty Liver Disease; MASLD: Metabolic Dysfunction-Associated Steatotic Liver Disease; NAFLD: Non-alcoholic Fatty Liver Disease; OR: Odds Ratio; and RR: Relative Risk.

**Table 4:** Key systematic reviews and meta-analyses on the association between heart failure (HF) and non-alcoholic fatty liver disease (NAFLD).

Author, Year, [Reference]	Meta-Analysis Characteristics	Median Follow-Up	Clinical Cardiovascular Outcome	HR or RR (95% CI)
Salah et al., 2022, [127]	Total Number of Studies: 5 Total Population: 1,433,066 NAFLD Population: 130,509	-	Incidence of HF	HR: 1.60 (1.24 to 2.05)
Li et al., 2022, [122]	Total Number of Studies: 6 Total Population: 10,979,967 NAFLD Population: 2,437,551	7 years	Incidence of HF	HR: 1.36 (1.16 to 1.58)
Alon et al., 2022, [46]	Total Number of Studies: 4 Total Population: 8,984,247 NAFLD Population: 2,465,243	4 years	Incidence of HF	HR: 1.61 (1.43 to 1.84)
Jaiswal et al., 2023, [128]	Total Number of Studies: 12 Total Population: 18,055,072 NAFLD Population: 2,938,753	6 years	Incidence of HF	RR: 1.43 (1.03 to 2.00)
Mantovani et al., 2023, [123]	Total Number of Studies: 11 Total Population: 11,242,231 NAFLD Population: 2,946,459	10 years	Incidence of HF	HR: 1.50 (1.34 to 1.67)

Abbreviations: CI: Confidence Interval; HF: Heart Failure; HR: Hazard Ratio; NAFLD: Non-alcoholic Fatty Liver Disease; and RR: Relative Risk.

## Data Availability

No new data were created or analyzed in this study. Data sharing is not applicable to this article.

## Abbreviations

The following abbreviations are used in this manuscript:

ACS	Acute coronary syndrome
ADMA	Asymmetric dimethyl arginine
AF	Atrial fibrillation
AIP	Atherogenic index of plasma
BMI	Body mass index
CAC	Coronary artery calcification
CARDIA	Coronary Artery Risk Development in Young Adults
CHD	Coronary heart disease
CI	Confidence interval
CIMT	Carotid intima-media thickness
CLMH	Cardiovascular–liver–metabolic health
CRP	C-reactive protein
CT	Computed tomography
CTCA	Computed tomography coronary angiography
CVD	Cardiovascular disease
E/A ratio	(EA) Early (E) to late atrial (A) transmitral flow velocity ratio
E/e′ ratio	Early transmitral flow velocity (E) to early diastolic mitral annular tissue velocity (e′) ratio
ECG	Electrocardiogram
FFA	Free fatty acids
FIB-4	Fibrosis-4 score
FLI	Fatty liver index
GGT	Gamma-glutamyl transferase
HbA1c	Glycated hemoglobin
HDL	High-density lipoprotein
HF	Heart failure
HFpEF	Heart failure with preserved ejection fraction
HFrEF	Heart failure with reduced ejection fraction
HOMA-IR	Homeostatic Model Assessment for Insulin Resistance
HR	Hazard ratio
IL	Interleukin
LA	Left atrium/atrial
LDL	Low-density lipoprotein
LV	Left ventricle/ventricular
LVH	Left ventricular hypertrophy
MAFLD	Metabolic dysfunction-associated fatty liver disease
MASLD	Metabolic dysfunction-associated steatotic liver disease
MASH	Metabolic-associated steatohepatitis
MetALD	Metabolic and alcohol-related/associated liver disease
MI	Myocardial infarction
NAFLD	Non-alcoholic fatty liver disease
NASH	Non-alcoholic steatohepatitis
NFS	NAFLD fibrosis score
NLRP3	NOD-like receptor family pyrin domain-containing protein 3 (inflammasome)
NO	Nitric oxide
OR	Odds ratio
OPERA	Oulu Project Elucidating Risk of Atherosclerosis
PNPLA3	Patatin-like phospholipase domain-containing protein 3
PTGS2	Prostaglandin-endoperoxide synthase 2
PDE11A	Phosphodiesterase 11A
RR	Risk ratio
SMD	Standardized mean difference
T2DM	Type 2 diabetes mellitus
TLR	Toll-like receptor
TM6SF2	Transmembrane 6 superfamily member 2
TMEM98	Transmembrane protein 98
TMAO	Trimethylamine N-oxide
TYMS	Thymidylate synthase
VEGF	Vascular endothelial growth factor
